# Commentary: Skilled Bimanual Training Drives Motor Cortex Plasticity in Children With Unilateral Cerebral Palsy

**DOI:** 10.3389/fnhum.2017.00297

**Published:** 2017-06-07

**Authors:** Deborah J. Serrien

**Affiliations:** School of Psychology, University of NottinghamNottingham, United Kingdom

**Keywords:** rehabilitation, pediatric, transcranial magnetic stimulation, neuroplasticity, hemiplegia

Unilateral spastic cerebral palsy (USCP) is a childhood-onset neurological disorder that is characterized by motor dysfunction to primarily one side of the body, reducing hand function of the affected hand, and bimanual coordination patterns (Staudt, 2010). To improve manual performance, effective motor rehabilitation techniques are crucial. However, optimization of the treatment methods is challenged by the heterogeneity of the clinical symptoms of children with USCP (Reid et al., 2015). Two main approaches currently exist: (1) constraint-induced movement therapy that involves physical restraint of the less affected upper extremity alongside practice of the more affected upper extremity, and (2) bimanual training therapy that consists of intensive practice of functional activities with both upper extremities. Therefore, the focus of these methods is distinct, although both use training intensity and progression of skill complexity as core characteristics of their programme. This rationale is based on the premise that practice with increased task difficulty drives plasticity of the motor system (Adkins et al., 2006).

In their study, Friel et al. (2016) examine the impact of bimanual training therapy upon manual performance and motor cortex plasticity in children with USCP. To this end, the authors compared structured skill training vs. unstructured hand use with each group of children having 90 h of bimanual therapy that were spread over 3 weeks of practice. The structured practice group received the Hand-Arm Bimanual Intensive Therapy (Charles and Gordon, 2006), which included the following key components: increase of task difficulty, practice of component movements that are part of a more complex task, and training of functional goals. In contrast, the unstructured practice group engaged in bimanual activities but did not train skillful movements or functional goals. Both groups were evaluated by outcome measures that quantified unimanual dexterity (Jebsen et al., 1969), bimanual performance (Krumlinde-Sundholm et al., 2007), and functional goal performance (Law et al., 1990). The authors further used single-pulse transcranial magnetic stimulation (TMS) to map the representation and excitability of the digit and wrist muscles of the affected hand at three time points: before, immediately after and 6 months after training. Combined, these measurements that assess cortical changes alongside motor skill improvements represent a powerful means to study how the brain responds to training.

The data showed that structured and unstructured training enhanced bimanual performance as well as hand dexterity of the affected hand; an improvement that was preserved at 6 month post-practice. Conversely, only the group that received structured training showed a significant increase in the size of the motor map of the affected hand and amplitude of the motor responses to TMS. This underlines the importance of skilled practice such as intricate bimanual activities as a critical factor for triggering cortical plasticity. However, despite improved hand function as a result of the intervention, little is known about the strengthening of the bimanual coordination pattern *per se*. In particular, although bimanual training therapy is aimed at augmenting the coordination of both upper limbs, the outcome measures focus on the functional improvement of the affected hand for tasks that require both hands rather than on measuring the process that characterizes the bimanual demands. In this respect, the coordinated use of both hands typically require an accurate spatio-temporal integration of each hand; an ability that develops with age (Fagard et al., 2001; Serrien et al., 2014; Babik and Michel, 2016) and that especially becomes apparent when performing daily-life activities during which the action of one hand is supported by a cooperative action from the other hand. Therefore, it is argued that the investigation of the bimanual synergy in relation to the individual task components could ascertain additional measurement outcomes, identify distinct time scales of motor learning (Serrien, 2008) and clarify interactions between levels of motor organization that occur as a result of rehabilitation.

The findings further revealed that the changes in cortical plasticity for the structured practice group were independent of the hemisphere of control, i.e., contralateral or ipsilateral to the affected hand. This suggests that both hemispheres responded to the bimanual training; an observation that contrasts with data from the constraint-induced movement therapy that have shown that functional gains are less pronounced in children with ipsilateral than contralateral control of the affected hand (Kuhnke et al., 2008). Taking into account that hand function in cerebral palsy is influenced by the nature of the corticospinal projections (Holmström et al., 2010), it is essential that training interventions are optimized across the pathology of USCP.

That the children who showed the strongest functional improvement also demonstrated the largest expansion of the cortical motor map points to neuroplasticity as a result of rehabilitation and is in line with the existence of reorganization mechanisms (Mackey et al., 2014). Thus, the present findings highlight the importance of studying brain-behavioral adaptations in children with USCP by using various experimental tools, tests, and time points during training interventions. This argument is especially relevant considering that the children who were part of the unstructured practice group improved their hand function with training but did not show modulations in the size of their cortical motor map within the time frame of investigation. Notwithstanding this clinical significance, corticospinal, and interhemispheric motor systems have not been fully studied in the developing brain. Therefore, detailing the properties of the motor circuits in childhood and adolescence will further enhance the evaluation of rehabilitation interventions for USCP.

Taken together, the study by Friel et al. (2016) provides exciting research that exposes the significance of skilled bimanual training for steering the interplay between neural changes and behavioral improvements in children with USCP. Further work that examines cortical plasticity across different motor systems and time scales alongside the development of measurements that assess coordination outcomes will be important to enhance insights into the neural recalibration that takes place in response to bimanual training. Due to advances in experimental acquisition and analysis techniques, future research will permit to detail how training with the aim to improve hand function in USCP shapes the brain and the mechanisms that support neuroplasticity.

## Author contributions

The author confirms being the sole contributor of this work and approved it for publication.

### Conflict of interest statement

The author declares that the research was conducted in the absence of any commercial or financial relationships that could be construed as a potential conflict of interest.
